# Noncytotoxic orange and red/green derivatives of DsRed-Express2 for whole-cell labeling

**DOI:** 10.1186/1472-6750-9-32

**Published:** 2009-04-03

**Authors:** Rita L Strack, Dibyendu Bhattacharyya, Benjamin S Glick, Robert J Keenan

**Affiliations:** 1 Department of Biochemistry and Molecular Biology, The University of Chicago, Chicago, Illinois 60637, USA; 2 Department of Molecular Genetics and Cell Biology, and Institute for Biophysical Dynamics, The University of Chicago, Chicago, Illinois 60637, USA

## Abstract

**Background:**

Whole-cell labeling is a common application of fluorescent proteins (FPs), but many red and orange FPs exhibit cytotoxicity that limits their use as whole-cell labels. Recently, a tetrameric red FP called DsRed-Express2 was engineered for enhanced solubility and was shown to be noncytotoxic in bacterial and mammalian cells. Our goal was to create derivatives of this protein with different spectral properties.

**Results:**

Building on previous studies of DsRed mutants, we created two DsRed-Express2 derivatives: E2-Orange, an orange FP, and E2-Red/Green, a dual-color FP with both red and green emission. We show that these new FPs retain the low cytotoxicity of DsRed-Express2. In addition, we show that these new FPs are useful as second or third colors for flow cytometry and fluorescence microscopy.

**Conclusion:**

E2-Orange and E2-Red/Green will facilitate the production of healthy, stably fluorescent cell lines and transgenic organisms for multi-color labeling studies.

## Background

Fluorescent proteins (FPs) are useful as whole-cell labels. For this purpose, FPs can be either monomeric or oligomeric. However, oligomeric FPs are often better for whole-cell labeling because they tend to be brighter and more photostable than their monomeric counterparts [1].

Even if an FP has desirable fluorescence properties, it may have limited utility as a cellular label due to cytotoxicity at high expression levels [2-4]. Cytotoxicity has been observed with many red and orange FPs in both bacterial and mammalian cells [5]. Recently, we described a tetrameric DsRed variant called DsRed-Express2 that is ideally suited to whole-cell labeling due to its minimal cytotoxicity, fast maturation, and high photostability [5]. To create DsRed-Express2, we mutated the surface of DsRed-Express (also known as DsRed.T1) [6] to decrease higher-order aggregation of the tetramers. These mutations allowed DsRed-Express2 to be well tolerated when expressed at high levels.

Here, we have modified the interior of DsRed-Express2 to create two additional FPs that are useful for whole-cell labeling. The first new FP, E2-Orange, exhibits orange fluorescence similar to that of previously described orange FPs [7-10]. E2-Orange matures quickly, and is substantially less cytotoxic and more photostable than other available orange FPs. The second new FP, E2-Red/Green, emits both red and green fluorescence, and can be distinguished from pure red or pure green FPs. E2-Orange and E2-Red/Green will be particularly useful for multi-color whole-cell labeling.

## Results and discussion

### An orange derivative of DsRed-Express2

Orange FPs can be useful alone, in two-color studies with green FPs, or in three-color studies with green and far-red FPs. The previously available orange FPs include the oligomeric Kusabira-Orange (KO) [9], a monomeric derivative of KO called mKO2 [8], and a monomeric orange variant of DsRed called mOrange2 [10]. To engineer an orange derivative of DsRed-Express2, we mutated the first residue of the chromophore, glutamine-66, to threonine. In mOrange, threonine at position 66 drives formation of a third heterocycle (oxazole ring) in the chromophore, leading to blue-shifted spectra relative to DsRed [7,11]. Introduction of the same Q66T mutation into DsRed-Express2 resulted in blue-shifted excitation and emission maxima, indicating that the same chromophore cyclization chemistry can occur in the DsRed-Express2 interior.

DsRed-Express2 + Q66T was then subjected to random mutagenesis to identify brightening mutations. We identified two such mutations, V71A and S179T. Both mutations produced modest increases in extinction coefficient and quantum yield, and the S179T mutation also accelerated maturation. These mutations were combined to yield the final orange variant, E2-Orange [GenBank: FJ498891].

**Figure 1:**
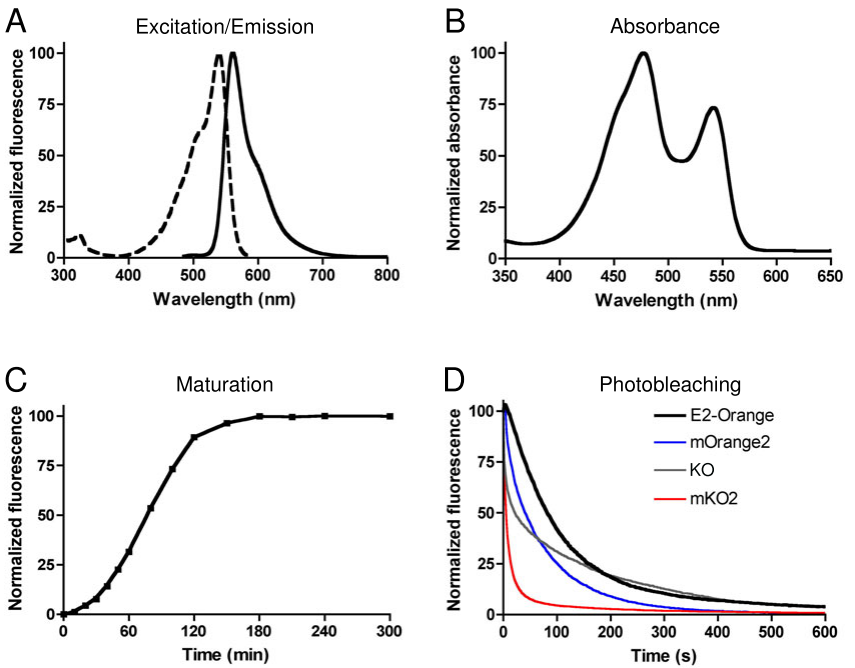
**Fluorescence properties of E2-Orange**. Shown are (A) excitation and emission and (B) absorbance spectra of E2-Orange. (C) Maturation kinetics of E2-Orange fluorescence. For these measurements the FPs were excited at 520 ± 10 nm excitation and emission was recorded at 560 ± 10 nm. (D) Photobleaching kinetics of E2-Orange (black line), mOrange2 (blue line), KO (gray line), and mKO2 (red line) during widefield fluorescence microscopy. Data were collected using a Texas Red filter set. For (C) and (D), data represent the mean of three independent measurements.

E2-Orange has excitation and emission maxima at 540 nm and 561 nm, respectively (Figure 1A). As with DsRed-Express2, a substantial fraction of the fully mature E2-Orange molecules contain a blue-absorbing and green-emitting chromophore (Figure 1B). However, excitation with blue light does not produce significant green fluorescence, presumably due to efficient intra-tetramer Förster resonance energy transfer (FRET). The presence of two chromophore species explains why E2-Orange has a lower extinction coefficient than other orange FPs when excited with yellow light (Table 1). When excited with blue light, E2-Orange is comparable in brightness to other orange FPs (data not shown).

**Table 1:** Properties of FPs.

Fluorescent Protein	Excitation/Emission maxima (nm)	Extinction coefficient (M^-1 ^cm^-1^)	Quantum yield	Relative brightness^a^	Maturation half-time (h)	Photobleaching half- time (s)^b^	pKa
DsRed-Express2^c^	554/591	35,600	0.42	0.41	0.7	64 ± 4	4.5
E2-Red/Green (green)	484/498	100,200	0.06	0.17	0.4	236 ± 8^d^	4.0
E2-Red/Green (red)	560/585	53,800	0.67	0.98	1.2	93 ± 3	4.5
E2-Orange	540/561	36,500	0.54	0.54	1.3	81 ± 3	4.5
mOrange2	549/563	56,300	0.49	0.75	4.5	40 ± 3	7.5
KO	548/560	72,800	0.55	1.1	3.8	21 ± 2	5.0
mKO2^c^	549/563	54,300	0.41	0.54	1.2	9 ± 1	5.0

Unless otherwise indicated, all measurements were carried out during the present study using standardized procedures.

^a ^Brightness was calculated as the product of extinction coefficient and quantum yield, and was normalized to a value of 1 for wild-type DsRed as calculated from Ref. 5.

^b ^Photobleaching half-times during widefield illumination are listed as mean ± s.e.m. for three independent replicates.

^c ^For DsRed-Express2 and mKO2, all data except the pK_a _measurements were taken from Ref. 5.

^d ^The photobleaching half-time for the green chromophore of E2-Red/Green cannot be compared directly to the photobleaching half-times of red chromophores because a different filter set was used.

E2-Orange matures quickly and is photostable (Table 1). Compared to previously available orange FPs, E2-Orange matures much faster than mOrange2 or KO and about as fast as mKO2, with a half-time of 1.3 h at 37°C (Figure 1C). We measured photostability with a simple assay involving a fixed illumination intensity [5], and found that E2-Orange is more photostable than any of the other orange FPs tested (Table 1, Figure 1D). E2-Orange has a pKa of 4.5, making it the least acid-sensitive of the orange FPs tested (Table 1). Thus, the fluorescence properties of E2-Orange are favorable for whole-cell labeling.

**Figure 2:**
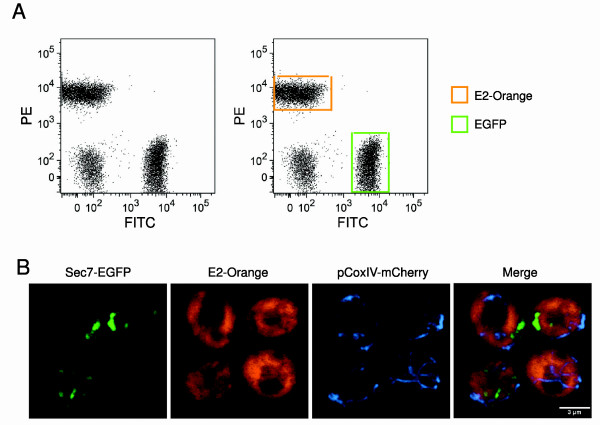
**E2-Orange is useful as a second or third color**. (A) Shown is a dot plot generated by flow cytometry of *S. cerevisiae *cells expressing E2-Orange, EGFP, or no FP. The three strains were grown separately, then pooled at equivalent cell concentrations and analyzed. Cells were excited using a 488-nm laser, and orange and green fluorescence signals were detected using PE and FITC filter sets, respectively. On the right are the same data with E2-Orange-expressing cells boxed in orange and EGFP-expressing cells boxed in green. (B) A three-color yeast strain was generated with GFP-labeled Golgi, E2-Orange-labeled cytosol, and mCherry-labeled mitochondria. Cells were imaged using confocal microscopy. E2-Orange and GFP were immediately resolvable, and E2-Orange and mCherry were resolvable after linear unmixing, yielding clear three color images as shown in the overlay.

To demonstrate the usefulness of E2-Orange in two-color labeling studies, the budding yeast *Saccharomyces cerevisiae *was transformed with vectors for high-level expression of either enhanced GFP (EGFP) or E2-Orange. By flow cytometry, these two populations of cells could be readily distinguished from each other and from cells not expressing an FP (Figure 2A).

To demonstrate the usefulness of E2-Orange as a third color in fluorescence microscopy, a *S. cerevisiae *strain was engineered to express cytosolic E2-Orange, plus Sec7-EGFP to mark the late Golgi with green fluorescence [12], plus pCoxIV-mCherry to mark mitochondria with moderately far-red fluorescence [6,7]. The EGFP and E2-Orange signals were easily resolvable, and the E2-Orange and mCherry signals could be separated using linear unmixing, yielding clear three-color images (Figure 2B). The need for linear unmixing indicates that FPs further red-shifted than mCherry (emission maximum = 610 nm) will be most useful for three-color imaging with E2-Orange and GFP.

### A red and green dual-color derivative of DsRed-Express2

An FP that has both red and green fluorescence can be a useful "third color" for flow cytometry and fluorescence microscopy [13]. Fully mature wild-type DsRed is a mixture of molecules with either red- or green-emitting chromophores, but excitation of the green chromophores yields red fluorescence due to intra-tetramer FRET [14]. Our previous identification of rapidly maturing DsRed variants led to discovery of the N42H mutation, which accelerates the maturation of wild-type DsRed more than 10-fold [6]. However, this mutation also increases the fraction of green-emitting chromophores, so that excitation with blue light now results in a substantial amount of green emission [6,15].

**Figure 3:**
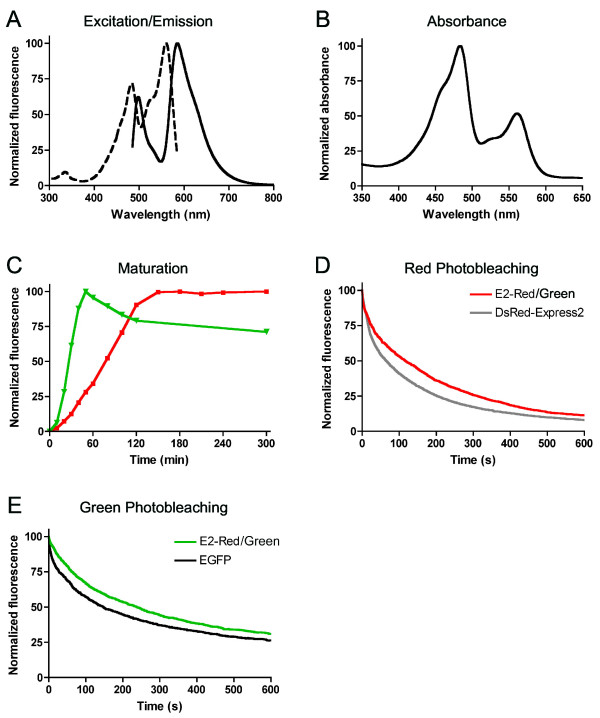
**Fluorescence properties of E2-Red/Green**. Shown are (A) excitation and emission and (B) absorbance spectra of E2-Red/Green. (C) Maturation kinetics of green (green line) and red (red line) fluorescence of E2-Red/Green. For these measurements the green species was excited with 480 ± 10 nm light and emission was recorded at 515 ± 10 nm, and the red species was excited with 540 ± 10 nm light and emission was recorded at 590 ± 10 nm. Also shown are photobleaching kinetics for the red fluorescence of E2-Red/Green and DsRed-Express2 (D) and for the green fluorescence of E2-Red/Green and EGFP (E). The red and green photobleaching measurements were recorded using Texas Red and Endow GFP filter sets, respectively.

We took advantage of the N42H mutation to create a dual-color red and green derivative of DsRed-Express2. First, DsRed-Express2 was mutated to revert chromophore-facing mutations that had previously been introduced to minimize green fluorescence (Table 2). The N42H mutation was then added to yield E2-Red/Green [GenBank: FJ498892]. When excited with blue light, E2-Red/Green emits strongly at both red and green wavelengths (Figure 3A). When excited with yellow light to produce red fluorescence, E2-Red/Green is as bright as wild-type DsRed and more than twice as bright as DsRed-Express2 (Table 1). The red and green chromophores of E2-Red/Green mature quickly with half-times of 1.2 h and 0.4 h, respectively (Figure 3C). Both chromophores are extremely photostable, even more so than the chromophores of DsRed-Express2 and EGFP (Figure 3D and 3E, Table 1).

**Table 2:** Substitutions made during the creation of E2-Red/Green and E2-Orange.

Construct	Residues differing from DsRed-Express2
E2-Red/Green^a^	Q42H, A44V, A217T, A219G
E2-Orange^b^	Q66T, V71A^c^, S179T^c^

^a ^GenBank Accession Number FJ498892

^b ^GenBank Accession Number FJ498891

^c ^These mutations were identified during random mutagenesis. All other mutations resulted from targeted mutagenesis.

**Figure 4:**
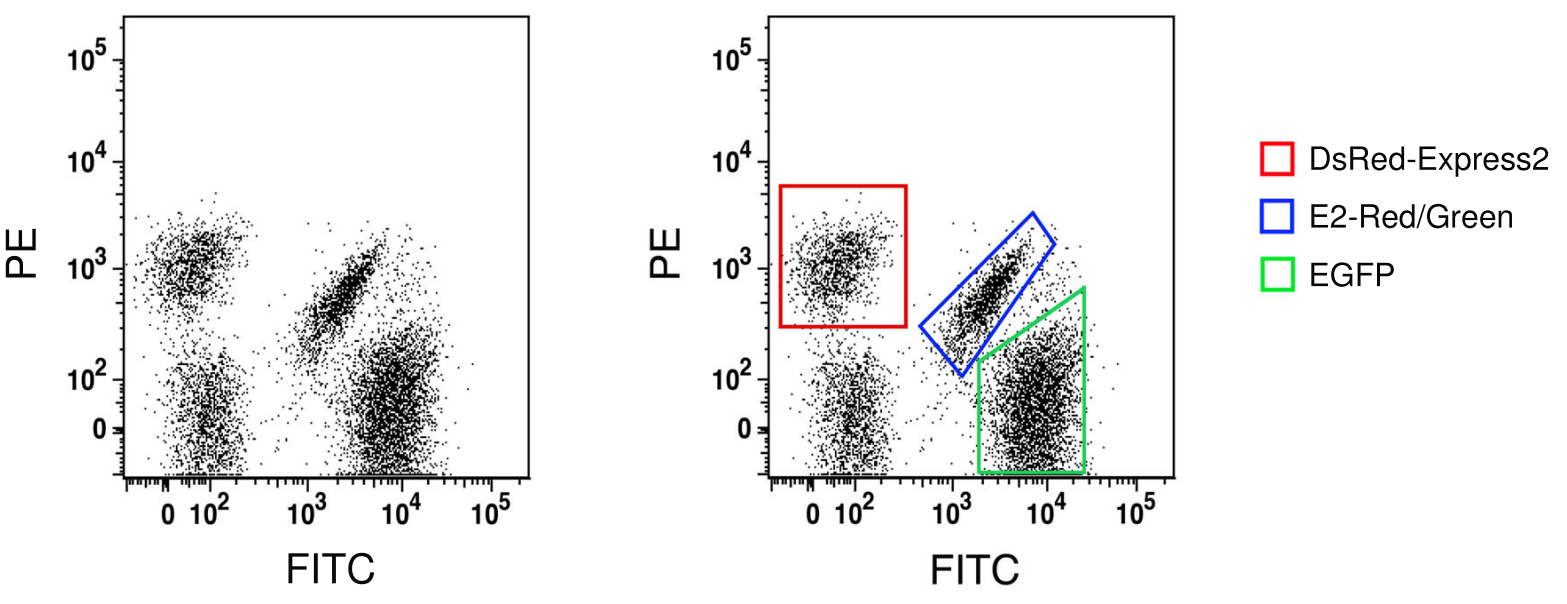
**E2-Red/Green is a useful third color for flow cytometry**. Shown is a dot plot generated by flow cytometry of *S. cerevisiae *cells expressing DsRed-Express2, E2-Red/Green, EGFP, or no FP. Cells were grown individually, pooled at equivalent cell concentrations, and analyzed by flow cytometry. Fluorescence was excited using a 488-nm laser, and red and green signals were detected using PE and FITC filter sets, respectively. On the right are the same data with DsRed-Express2-expressing cells boxed in red, E2-Red/Green-expressing cells boxed in blue, and EGFP-expressing cells boxed in green.

To show that E2-Red/Green can be used as a third marker in conjunction with a green and a red FP, *S. cerevisiae *cells expressing DsRed-Express2, EGFP, E2-Red/Green, or no FP were grown separately and then pooled. This mixture was analyzed by flow cytometry with a 488-nm excitation laser. The three populations of fluorescent cells could be readily distinguished from one another and from the population of cells not expressing an FP (Figure 4).

### The new FPs are soluble and show low cytotoxicity and low phototoxicity in bacteria

High-level expression in bacteria is the basis of convenient assays for FP solubility, cytotoxicity, and phototoxicity [5]. We used this approach to compare the new FPs to DsRed-Express2 and to previously available orange FPs. One complication was that human codon-optimized KO is expressed poorly in bacteria, presumably due to unfavorable mRNA secondary structure near the start codon [16,17]. This problem was overcome by applying a method that had previously been used to optimize bacterial expression of DsRed-Express2 [5]. We screened bacteria expressing a library of modified KO genes that contained random combinations of the synonymous codons for amino acids 2–6. A sequence yielding colonies with bright fluorescence was designated KO*. Predicted secondary structures near the 5' ends of the KO and KO* mRNA sequences were compared using the program mfold [18]. The KO mRNA contained a predicted stable stem-loop involving the ribosomal binding site, and this stem-loop was disrupted in the KO* mRNA (data not shown). This change may account for the stronger bacterial expression of KO*.

**Figure 5:**
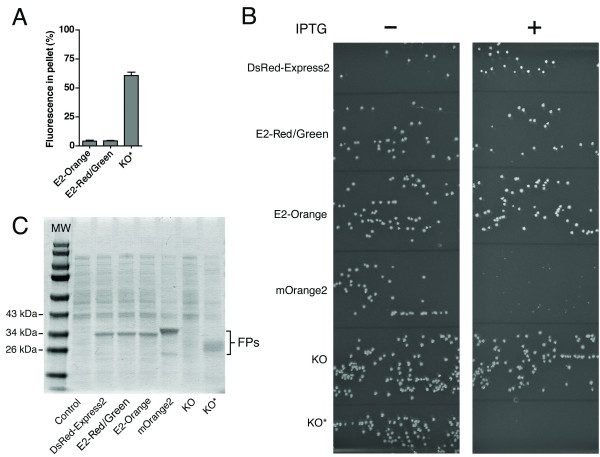
**E2-Orange and E2-Red/Green do not form higher-order aggregates and are noncytotoxic to bacteria**. (A) To assay higher-order aggregation of oligomeric FPs, the percent fluorescence in the pellet fraction of a lysate from *E. coli *cells expressing E2-Orange, E2-Red/Green, or KO* was measured for eight independent replicates. Error bars represent s.e.m. (B) To measure bacterial cytotoxicity of FPs, *E. coli *DH10B cells harboring the pREP4 repressor plasmid were transformed with pQE-60NA encoding DsRed-Express2, E2-Red/Green, E2-Orange, mOrange2, KO, or KO*. Equal volumes of transformation mixtures were plated onto adjacent sectors of plates under either repressing (no IPTG) or derepressing (1 mM IPTG) conditions. Large colonies under derepressing conditions (right panel) indicate low cytotoxicity. (C) Quantitation of FP expression under derepressing conditions. Cells were grown to an OD_600 _of ~ 0.6 and then treated with 1 mM IPTG for 4 h. Whole-cell lysates were separated using SDS-PAGE followed by staining with Coomassie Blue. Control cells were transformed with empty pQE-60NA.

To confirm that the new FPs retain the high solubility of DsRed-Express2, we used a bacterial lysis and centrifugation assay in which aggregated oligomeric FPs are found in the pellet [5,6]. As with DsRed-Express2, only 3–5% of the total fluorescence signal was found in the pellet with E2-Orange and E2-Red/Green (Figure 5A). By contrast, with KO*, > 50% of the fluorescence signal was in the pellet (Figure 5A), indicating that this protein undergoes higher-order aggregation.

To test whether the new FPs retain the low bacterial cytotoxicity of DsRed-Express2, we examined colony size for *E. coli *cells expressing high levels of various FPs [5]. This experiment employed a regulatable promoter in which expression was derepressed using isopropyl-β-D-thiogalactopyranoside (IPTG). In the absence of IPTG, all of the colonies were large, as expected (Figure 5B). When IPTG was included in the medium, large colonies were seen for cells expressing DsRed-Express2, E2-Orange, or E2-Red/Green, but much smaller colonies were seen for cells expressing mOrange2 or KO*, indicating that the latter two proteins are cytotoxic (Figure 5B). We previously documented that mKO2 is also cytotoxic in bacteria [5]. SDS-PAGE of whole-cell lysates confirmed that all of the FP genes except the original KO were expressed at comparable levels (Figure 5C). KO did not cause cytotoxicity in the presence of IPTG (Figure 5B) due to very low expression (Figure 5C). These data indicate that like DsRed-Express2, E2-Orange and E2-Red/Green exhibit unusually low cytotoxicity in bacteria.

**Figure 6:**
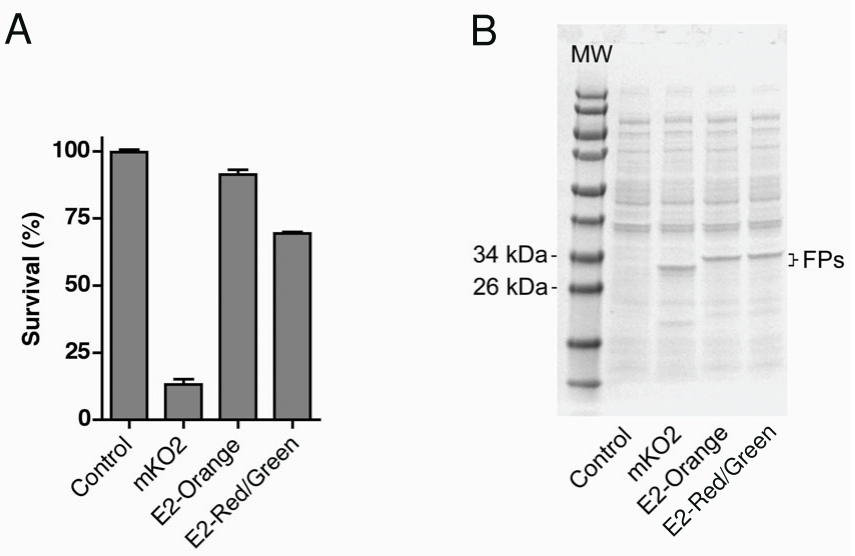
**E2-Orange and E2-Red/Green show relatively mild phototoxicity**. (A) *E. coli *cells were treated for 4 h with 1 mM IPTG to express either no FP (Control) or the indicated FP. Cells were then illuminated through a Texas Red (535–585 nm) filter for 15 min. In parallel, identical samples were not illuminated. Cells were then plated and grown overnight, and the percent survival was calculated based on colony number for the illuminated versus non-illuminated samples. Error bars represent s.e.m. (B) Quantitation of FP expression under the conditions of the phototoxicity experiment. Immediately before light treatment, aliquots of cells were taken for expression analysis. Whole-cell lysates were separated using SDS-PAGE followed by staining with Coomassie Blue.

Another experimentally relevant FP characteristic is phototoxicity during prolonged illumination. We used a bacterial cell survival assay [5] to measure the phototoxicity of E2-Orange, E2-Red/Green, and mKO2 during illumination through a Texas Red filter. This assay was not technically feasible with mOrange2 or KO* due to their slow maturation rates (Table 1). Control cells not expressing an FP showed nearly 100% survival (Figure 6A). E2-Orange and E2-Red/Green showed low to moderate phototoxicity with 91% and 69% survival, respectively. mKO2 showed high phototoxicity, with only 13% survival under the same conditions. These differences in phototoxicity were not due to different expression levels (Figure 6B). We conclude that E2-Orange and E2-Red/Green are likely to exhibit relatively mild phototoxicity during live-cell imaging.

### The new FPs show low cytotoxicity in HeLa cells

**Figure 7:**
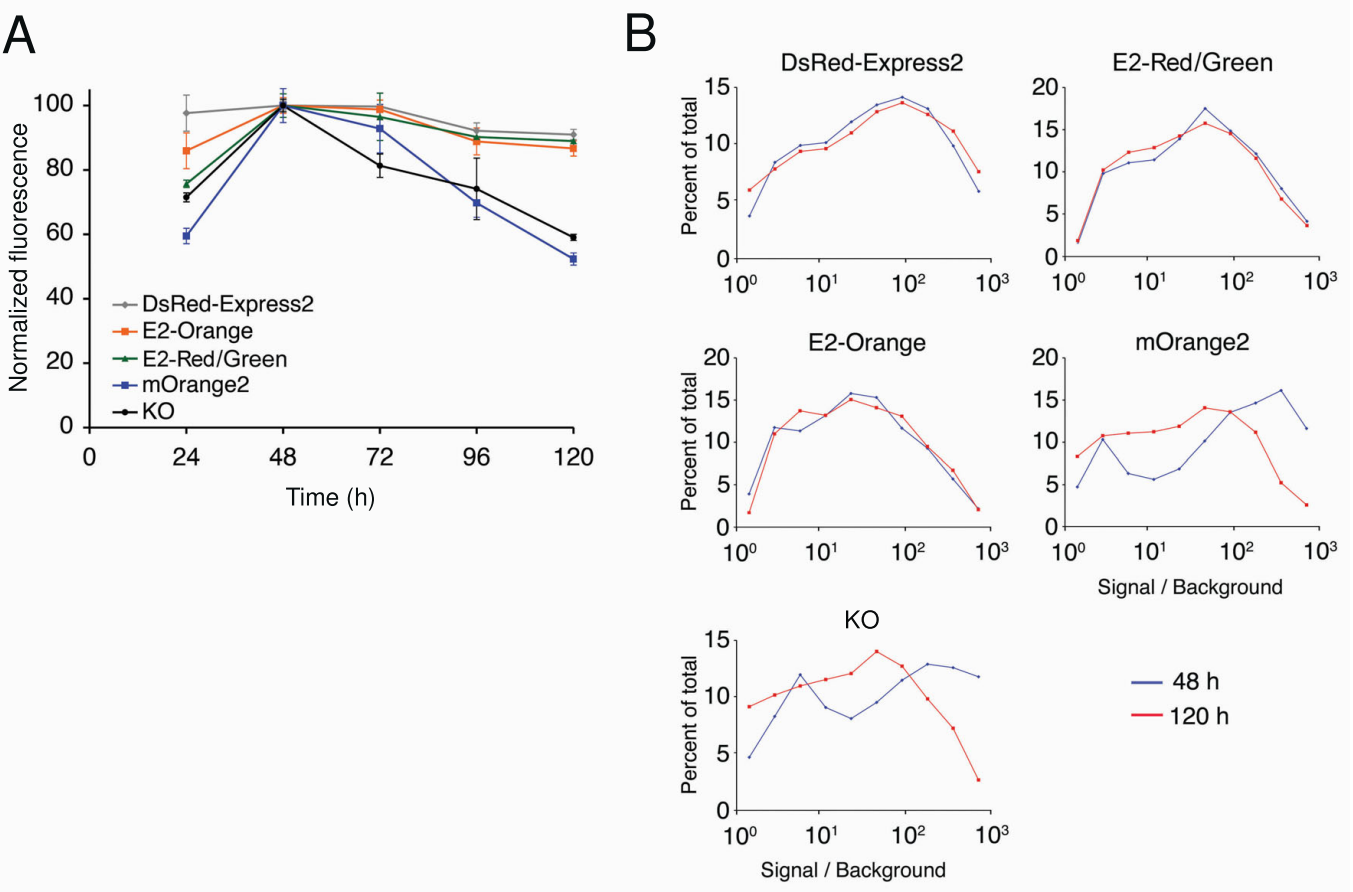
**E2-Orange and E2-Red/Green are noncytotoxic to HeLa cells under conditions of standard high-level expression**. (A) HeLa cells were transiently transfected in 24-well plates for constitutive high-level expression of the indicated FP. Three wells per FP per day were analyzed by flow cytometry, in parallel with untransfected cells, and the average brightness of the viable fluorescent cells was measured. The highest signal for a given FP was normalized to 100 units. Error bars represent s.e.m. (B) Fluorescence intensity distributions for the same data were analyzed for 48 h (blue) and 120 h (red) post-transfection. Each data point is a binned value that represents the percentage of cells with fluorescence in a range centered about the data point.

To determine whether the DsRed-Express2 derivatives retain low cytotoxicity in mammalian cells, a fluorescence maintenance assay was performed [5]. HeLa cells were transiently transfected to express DsRed-Express2, E2-Orange, E2-Red/Green, mOrange2, or KO under control of the strong CMV promoter, and the average brightness of fluorescent cells was monitored for five days using flow cytometry (Figure 7A). As previously observed, cells expressing DsRed-Express2 remained bright over the course of the experiment. Similarly, for cells expressing E2-Orange or E2-Red/Green, average cellular brightness was maximal at 48 h post-transfection and had decreased by < 10% at 120 h post-transfection. By contrast, for cells expressing mOrange2 or KO, average cellular brightness had decreased at 120 h by 40–50%. The source of this decrease can be seen by comparing the flow cytometry data from 48 h and 120 h. We assigned cells to groups, each of which represented a range of brightness values, and then plotted the percentage of cells in each group (Figure 7B). At 48 h, the brightness of the transfected cells spanned from background fluorescence up to about 1000-fold over background. At 120 h, cells expressing high levels of DsRed-Express2, E2-Orange, or E2-Red/Green were detected with unchanged frequencies, but cells expressing high levels of mOrange2 or KO were depleted from the population (Figure 7B). A similar loss of highly expressing cells was documented previously for mKO2 [5]. The combined data indicate that DsRed-Express2 and its derivatives are noncytotoxic even at high expression levels, whereas previously available orange FPs show considerable cytotoxicity in mammalian cells.

## Conclusion

We predicted that it should be possible to modify the interior of DsRed-Express2 while preserving the optimized surface, thereby generating new noncytotoxic FPs with a variety of spectral properties [5]. The current study verifies that prediction. We incorporated mutations that had been reported to change the DsRed emission either to orange [7] or to a mixture of red and green [6,15]. The resulting DsRed-Express2 derivatives, E2-Orange and E2-Red/Green, have favorable fluorescence properties including brightness, fast maturation, and high photostability. Importantly, these new FPs maintain the low cytotoxicity of DsRed-Express2 in bacterial and mammalian cells.

E2-Orange outperforms other available orange FPs by several criteria (Table 1). For comparison, mOrange2 is photostable, but matures slowly and is very acid-sensitive [10] with a pKa of 7.5. mKO2 matures as quickly as E2-Orange, but is photolabile and phototoxic. Of the previously available FPs, KO is probably the best suited to whole-cell labeling because its slow maturation is offset by high brightness and reasonable photostability. Indeed, KO has been used to label murine hematopoietic stem cells [19] and to generate systemically labeled transgenic pigs [20]. How can we reconcile these results with the clear cytotoxicity of KO in our assays? We propose that reports of stable expression of KO and other cytotoxic FPs [21-23] reflect a threshold effect in which a cytotoxic FP is tolerated below a certain expression level. Thus, even if a cytotoxic FP is intrinsically bright, the observed signal may be relatively weak due to selection for low expression, and this low expression may cause residual cytotoxicity. By contrast, high-level expression of E2-Orange is likely to be maintained with little detrimental effect to the cells.

E2-Red/Green can be used as a "third color" in combination with existing green and red FPs in cases where three distinct cell populations each express a single type of FP. This type of application was suggested by Verkhusha and colleagues, who described a novel GFP variant, R10-3, in which a small fraction of the molecules generate a red chromophore [13]. E2-Red/Green should be easier to use as a dual-color cell label because its red emission is approximately 100-fold greater than that of R10-3. As an example of a potential application of E2-Red/Green, a standard 488-nm laser efficiently excites EGFP, DsRed-Express2, and E2-Red/Green, allowing three cell populations to be distinguished by flow cytometry. For fluorescence microscopy applications, images from green and red channels can be overlaid to distinguish cells expressing E2-Red/Green from those expressing either a pure green or a pure red FP. In this case, the cells expressing E2-Red/Green will appear yellow in the overlaid images, while the other cells will appear either red or green.

Because E2-Orange and E2-Red/Green are tetramers, they are useful for whole-cell labeling rather than for making fusion proteins. Further modifications of the chromophore environment should enable us to generate additional whole-cell labels. For example, a far-red derivative of DsRed-Express2 would be ideal for whole-animal imaging, and for multi-color labeling of cell populations in conjunction with E2-Orange.

## Methods

### Characterization of FPs

EGFP was obtained from Clontech. mOrange and mCherry were provided by R. Tsien (University of California at San Diego), and mOrange was modified by site-directed mutagenesis to create mOrange2. mKO2 was obtained as previously described [5]. Humanized KO was obtained by gene synthesis (GenScript). To create KO*, we replicated an earlier strategy that was used to enhance the bacterial expression of DsRed-Express2 [5]. Specifically, PCR of the humanized KO gene was performed using forward primers representing all possible synonymous codons for residues 2–6 (a mixture of 5'-TAGTAACCATGGTNTCNGTNATHAARCCCGAGATGAAGATGAAGTACTTC-3' and 5'-TAGTAACCATGGTNAGYGTNATHAARCCCGAGATGAAGATGAAGTACTTC-3'). This PCR product was then digested with NcoI and NotI and subcloned into pQE-60NA [5], transformed into *E. coli *strain XL-1 Blue, and plated onto LB + 100 μg/ml ampicilin lacking IPTG. After 12 h growth at 37°C, cells were placed at room temperature for 10 h to allow for leaky expression. Colonies were then screened with the slide projector assay as previously described [5,6] using a 520 ± 20 excitation filter and a 550-nm longpass emission filter. Brightly fluorescent colonies were restreaked onto LB + 100 μg/ml ampicilin, and the brightest variant was sequenced and designated KO*. The sequence of codons 1–6 in KO* is 5'-ATGGTCAGTGTGATAAAA-3'. Secondary structure analysis of KO and KO* was carried out using mfold [18]. The nucleotide sequences used for this analysis began 15 bp upstream of the ribosomal binding site and ended at codon 11 of the protein coding regions.

FPs were expressed, purified, and characterized for extinction coefficient and quantum yield as previously described [5,6,24]. The quantum yield of green chromophores was obtained using fluorescein in 0.1 M NaOH as a reference. The aggregation assay was carried out as previously described [5]. Photobleaching assays were carried out as previously described [5] using a Zeiss AxioPlan2 epifluorescence microscope with a 100-W mercury arc lamp, a 40× 0.75-NA air objective, and a Texas Red (535–585 nm excitation) or Endow GFP (450–490 nm excitation) filter set (Chroma).

pK_a _values were measured as follows. Purified FP was buffer exchanged and concentrated to 2 mg/ml into buffer containing 5 mM Na^+ ^HEPES pH 8.0 and 100 mM NaCl. Aliquots were then adjusted to a final pH of 3.5–10.0 in 0.5 pH unit increments by adding 5 μl of a 1 M adjustment buffer to 45 μl of the FP sample. The adjustment buffers were Na^+ ^citrate (pH 3.5–5.5), Bis-Tris (pH 6–6.5), Na^+ ^HEPES (pH 7.0–8.5), and Na^+ ^CAPSO (pH 9.0–10.0). Fluorescence measurements were carried out on a Tecan Safire^2 ^plate reader. Orange FPs were excited with 520 ± 10 nm light, and emission was recorded at 560 ± 10 nm. Red FPs were excited with 540 ± 10 nm light, and emission was recorded at 590 ± 10 nm. Green FPs were excited with 480 ± 10 nm light, and emission was recorded at 515 ± 10 nm. Fluorescence values for a given FP were normalized to the highest observed emission, and the pK_a _was determined to be the point at which the fluorescence was half of the maximal signal.

Maturation experiments were carried out as previously described [24]. Briefly, *E. coli *DH10B cells harboring a given FP in the pQE-81 vector were treated with 2 mM IPTG for 15 min followed by inhibition of translation with 30 μg/ml kanamycin plus 17 μg/ml tetracyline. Fluorescence measurements were then recorded at regular time intervals using a Tecan Safire^2 ^plate reader. For this assay, orange FPs were excited with 520 ± 10 nm light, and emission was recorded at 560 ± 10 nm. Red FPs were excited with 540 ± 10 nm light, and emission was recorded at 590 ± 10 nm. Green FPs were excited with 480 ± 10 nm light, and emission was recorded at 515 ± 10 nm.

### Library construction and screening

Targeted mutations were introduced into the DsRed-Express2 gene by overlap extension PCR [25]. Combinatorial gene libraries with random mutations were built by error-prone PCR [26]. The PCR products were subcloned into pQE-60NA, transformed into *E. coli *strain DH10B, and analyzed for brightness and color using the slide projector assay.

### Bacterial cytotoxicity and phototoxicity assays

For the *E. coli *colony size assay, DH10B cells harboring the pREP4 repressor plasmid were transformed with pQE-60NA encoding the relevant FP. Equal volumes of cell mixture from the same transformation tube were plated either on an LB + 50 μg/ml carbenicillin + 30 μg/ml kanamycin plate, or on a similar plate containing 1 mM IPTG. Colonies were photographed after growth for 14 h at 37°C. Phototoxicity was assayed as previously described [5].

### Fluorescence microscopy and flow cytometry of yeast

To generate the three-color yeast strain, mCherry was subcloned into a plasmid carrying a yeast CoxIV presequence (pCoxIV) [6], and this plasmid was transformed into *S. cerevisiae *strain JK9-3da expressing Sec7-3xGFP [12]. This two-color strain was then transformed with a YIplac204 [27] derivative in which E2-Orange has been subcloned between the *S. cerevisiae TPI1 *promoter and the *CYC1 *terminator [28]. The resulting three-color cells were grown in quasi-synthetic dropout (QSD) media lacking leucine [12] and imaged using a Leica SP5 confocal microscope with a 63× oil objective. Linear unmixing was performed using Leica application suite (LAS-AF) software version 2.0, and image processing was performed using ImageJ .

Yeast strains expressing a single FP were generated by transforming JK9-3da cells with a YIplac204 derivative containing the desired FP subcloned between the *S. cerevisiae TPI1 *promoter and the *CYC1 *terminator. Labeled yeast or untransformed control cells were grown individually in QSD media and pooled based on equivalent OD_600 _units. An LSRII flow cytometer (BD Biosciences) with a 488-nm laser was used with either FITC (515/50) or PE (585/15) emission filter sets to analyze cells. All data were processed using FloJo software (Treestar).

### HeLa cell cytotoxicity assay

Transient transfection of HeLa cells and subsequent analysis by flow cytometry were carried out as previously described [5].

### Availability of E2-Orange and E2-Red/Green

These plasmids can be obtained by request from Robert Keenan bkeenan@uchicago.edu.

## Authors' contributions

RLS engineered E2-Orange and E2-Red/Green, and carried out all experiments. DB assisted with the mammalian tissue culture experiments. RLS, BSG, and RJK participated in conception of the project, design of the study, and manuscript preparation. All authors read and approved the final manuscript.
